# Dedifferentiated liposarcoma with low-grade fibromyxoid sarcoma and inflammatory myofibroblastic tumor: A case report and literature review

**DOI:** 10.1097/MD.0000000000043806

**Published:** 2025-08-15

**Authors:** Jing Huang, Yuzhen Huang, Xiaoyu Chen, Lixia Zeng, Yun Ma

**Affiliations:** a Department of Pathology, Guangxi Medical University Cancer Hospital, Nanning, China.

**Keywords:** DDLPS, dedifferentiated liposarcoma, inflammatory myofibroblastic tumor, low-grade fibromyxoid sarcoma, *MDM2* amplification

## Abstract

**Rationale:**

Dedifferentiated liposarcoma (DDLPS) is a subtype of sarcoma that originates from atypical lipomatous tumor/well-differentiated liposarcoma and undergoes varying degrees of dedifferentiation. DDLPS can occur either concurrently with or after atypical lipomatous tumor/well-differentiated liposarcoma, with the dedifferentiated component predominantly consisting of high-grade sarcomas. Notably, only approximately 10% of DDLPS cases present as purely low-grade sarcomas. Cases exhibiting characteristics of both low-grade fibromyxoid sarcoma (LGFMS) and inflammatory myofibroblastic tumor (IMT)-like features have not been reported. Moreover, histopathologic overlap with other mesenchymal neoplasms, particularly IMT and LGFMS, poses significant diagnostic challenges. This morphologic similarity frequently causes diagnostic confusion with critical therapeutic implications, given the markedly divergent management strategies for these entities. This study aims to clarify the pathologic features and differential diagnosis of DDLPS to improve diagnostic recognition among pathologists.

**Patient concerns:**

A 60-year-old man presented with a 1-month history of progressive enlargement of a painless left abdominal mass. Associated constitutional symptoms, including fever and unintentional weight loss, were absent.

**Diagnoses:**

Dedifferentiated liposarcoma (FNCLCC grade 2) featuring well-differentiated, IMT-like, and LGFMS-like components. This conclusion was confirmed by the demonstration of *MDM2* amplification via fluorescence in situ hybridization.

**Interventions:**

The patient underwent complete resection. Postoperatively, no adjuvant therapy was administered. Surveillance comprised quarterly abdominothoracic computed tomography scans for the first 2 postoperative years, per institutional protocol.

**Outcomes:**

At the 1-year follow-up, surveillance imaging showed no evidence of local recurrence or distant metastasis. The patient remained asymptomatic with preserved renal and gastrointestinal function.

**Lessons:**

This case underscores critical clinical lessons: diagnostic vigilance is paramount when assessing sarcomas with mixed morphological patterns, given that DDLPS may closely mimic IMT or LGFMS. To prevent misdiagnosis, a systematic diagnostic approach radiological, immunohistochemistry, and confirmatory molecular testing is essential. Therapeutic management relies on complete surgical resection, with adjuvant therapy decisions guided by multidisciplinary evaluation. Long-term surveillance remains necessary due to recognized risks of late recurrence in retroperitoneal DDLPS, mandating sustained follow-up.

## 1. Introduction

In the fifth edition of World Health Organization Classification of Tumors of Soft Tissue and Bone,^[1]^ liposarcomas are categorized into 5 subtypes, namely atypical lipomatous tumor/well-differentiated liposarcoma (ALT/WDLPS), dedifferentiated liposarcoma (DDLPS), myxoid liposarcoma, pleomorphic liposarcoma, and myxoid pleomorphic liposarcoma. DDLPS is defined as a non-lipogenic sarcoma that originates from ALT/WDLPS and undergoes dedifferentiation into varying histological grades within primary or recurrent tumors. High-grade dedifferentiation includes pleomorphic undifferentiated sarcoma, myxofibrosarcoma, or malignant peripheral nerve sheath tumors. Approximately 10% of DDLPS cases demonstrate low-grade dedifferentiation with morphological patterns resembling fibromatosis or meningioma-like whorled structures.^[2]^ Reports of low-grade dedifferentiation resembling low-grade fibromyxoid sarcoma (LGFMS) or inflammatory myofibroblastic tumor (IMT) are rare, with cases exhibiting dedifferentiation into 2 distinct low-grade tumor morphologies being even more exceptional.

Accurate pathological diagnosis of DDLPS is paramount for optimal patient management. Surgical resection, aiming for R0 or R1 margins, constitutes the primary treatment and is associated with superior outcomes compared to R2 resection, although achieving complete resection is often anatomically constrained in the retroperitoneum.^[3]^ Consequently, adjuvant therapies are frequently employed. Preoperative radiotherapy improves local control in grade 1 to 2 DDLPS,^[4]^ whereas chemotherapy (doxorubicin with or without ifosfamide) has demonstrated no significant improvement in overall survival and is associated with considerable toxicity, notably hematological adverse events.^[5]^ Emerging targeted agents, particularly inhibitors of characteristic pathways such as MDM2 and CDK4 where CDK4 serves as a prognostic biomarker,^[6]^ along with other candidates,^[7]^ offer promising therapeutic alternatives. Thus, precise pathological identification enables patients with DDLPS to access and potentially benefit from this evolving spectrum of combined-modality approaches.

Herein, we present a case of DDLPS exhibiting histological features resembling both LGFMS and IMT. This article discusses the clinical, morphological, and molecular characteristics of this case, corroborated by a literature review.

## 2. Case report

**Figure 1.**
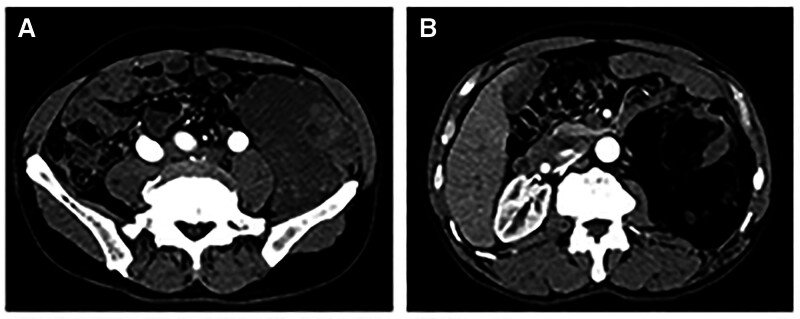
CT scans: solid mass shadow with uneven density in the left posterior abdominal cavity (A); mixed-signal shadow with predominantly fat components (B). CT = computed tomography.

A 60-year-old man presented to an external hospital with persistent left-sided abdominal swelling for over 1 month. A contrast-enhanced computed tomography scan of the entire abdomen indicated a mass in the left retroperitoneal region extending into the colonic wall, exhibiting heterogeneous density and irregular enhancement. Above the lesion, another area resembling a mass primarily comprising fat with mixed density was observed, consisting of patchy, linear, and nodular soft tissue densities. The solid components displayed uneven enhancement, whereas the fatty components showed no enhancement. The lesion borders were indistinct, exerting compression on the adjacent bowel loops and the left kidney with unclear demarcation from the descending colon (Fig. 1A, B). During an open radical resection, the tumor involved the retroperitoneum, left posterior abdominal cavity, and part of the colonic wall/mesocolon, with diameters of 20, 7, and 17 cm, respectively.

**Figure 2.**
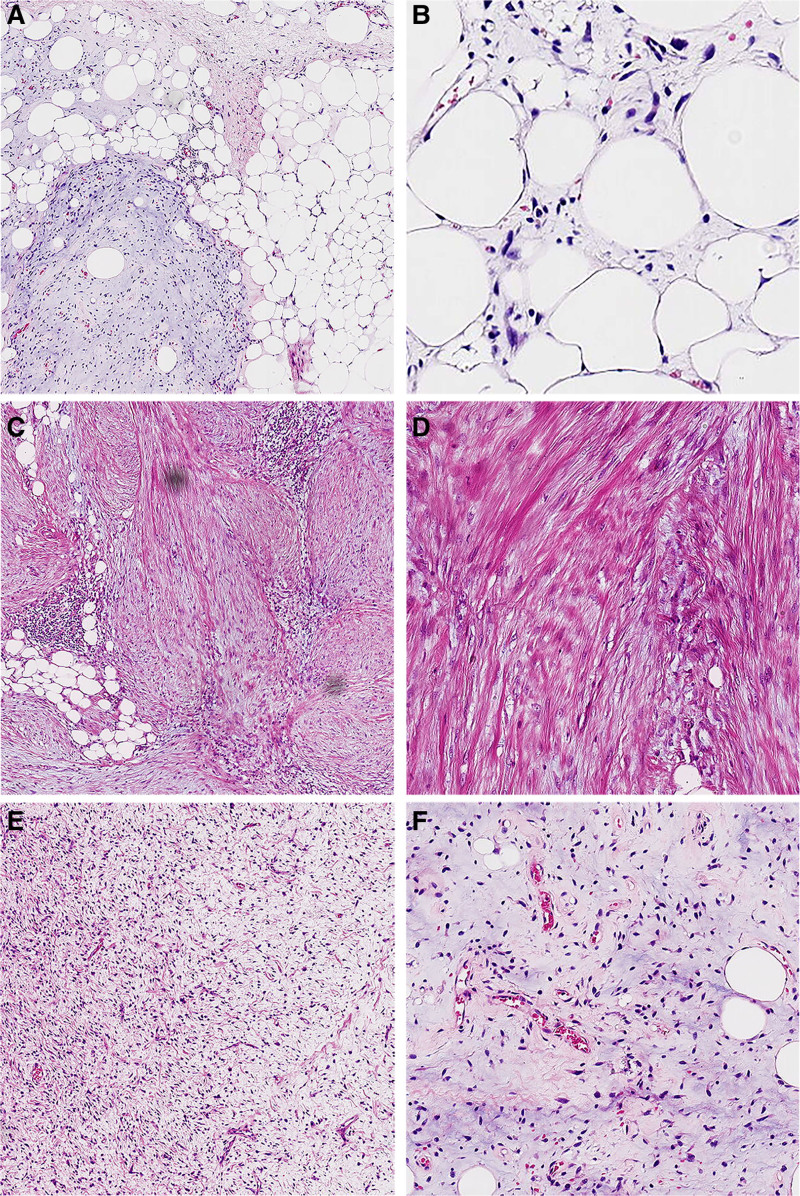
(A) H&E staining reveals WDLPS characterized by adipocytes of varying sizes arranger in lamellar and lobular patterns, featuring deeply stained nuclei and focal areas with mucinous stroma. (B) High power image of atypical mesenchymal stromal cells with enlarged nuclei. (C) IMT-like region showing solid, interstitial sparing edema, lymphocyte aggregation and infiltration. (D) High power image showing plump spindle-shaped myofibroblasts with small nucleoli. (E) LGFMS-like area showing fibrocollagenous sclerosis (lower left) and areas rich in mucus (upper right). (F) High power image showing tumor cells with mild morphology, bow-shaped thin-walled blood vessels in the mucinous areas. H&E = hematoxylin and eosin, IMT = inflammatory myofibroblastic tumor, LGFMS = low-grade fibromyxoid sarcoma, WDLPS = well-differentiated liposarcoma.

Tumor histomorphology predominantly demonstrated 3 structures as follows: ALT/WDLPS, LGFMS-like tumors, and IMT-like morphologies. The boundaries among these structures were distinctly demarcated. ALT/WDLPS, observed in the left posterior abdominal cavity, exhibited scattered patches and lobules of relatively mature adipocytes of varying sizes. Adjacent to the occasional vacuolated fat progenitor cells, non-atypical stromal cells exhibited enlarged nuclei and deep staining. Focal areas displayed mucinous stroma (Fig. 2A, B). However, tumor located in the colonic wall/ mesocolon predominantly exhibit a distinct transition from adipose regions to densely organized bundle-like structures. The tumor is more solid, focal areas with reduced cell density displayed enhanced interstitial collagen fibers with moderate tumor cell density, characterized by spindle-shaped cells featuring vesicular nuclei and 1 to 2 prominent nucleoli. Additionally, a few mitotic figures and focal infiltration of inflammatory cells, accompanied by lymphocyte aggregates, were observed (Fig. 2C, D). We conclude that this lesion exhibits characteristics indicative of IMT. In the resected retroperitoneal tumor, we identified a distinct morphological pattern characterized by alternating fibrocollagenous and myxoid areas, reminiscent of LGFMS. Tumor cells within this region exhibited ovoid, short spindle-shaped, or stellate morphologies with relatively moderate appearances. Tumor cells are disorganized in the collagenous fiber-rich regions, while in the myxoid areas, characteristic thin-walled vessels with arched and branching patterns are observed (Fig. 2E, F).

**Figure 3.**
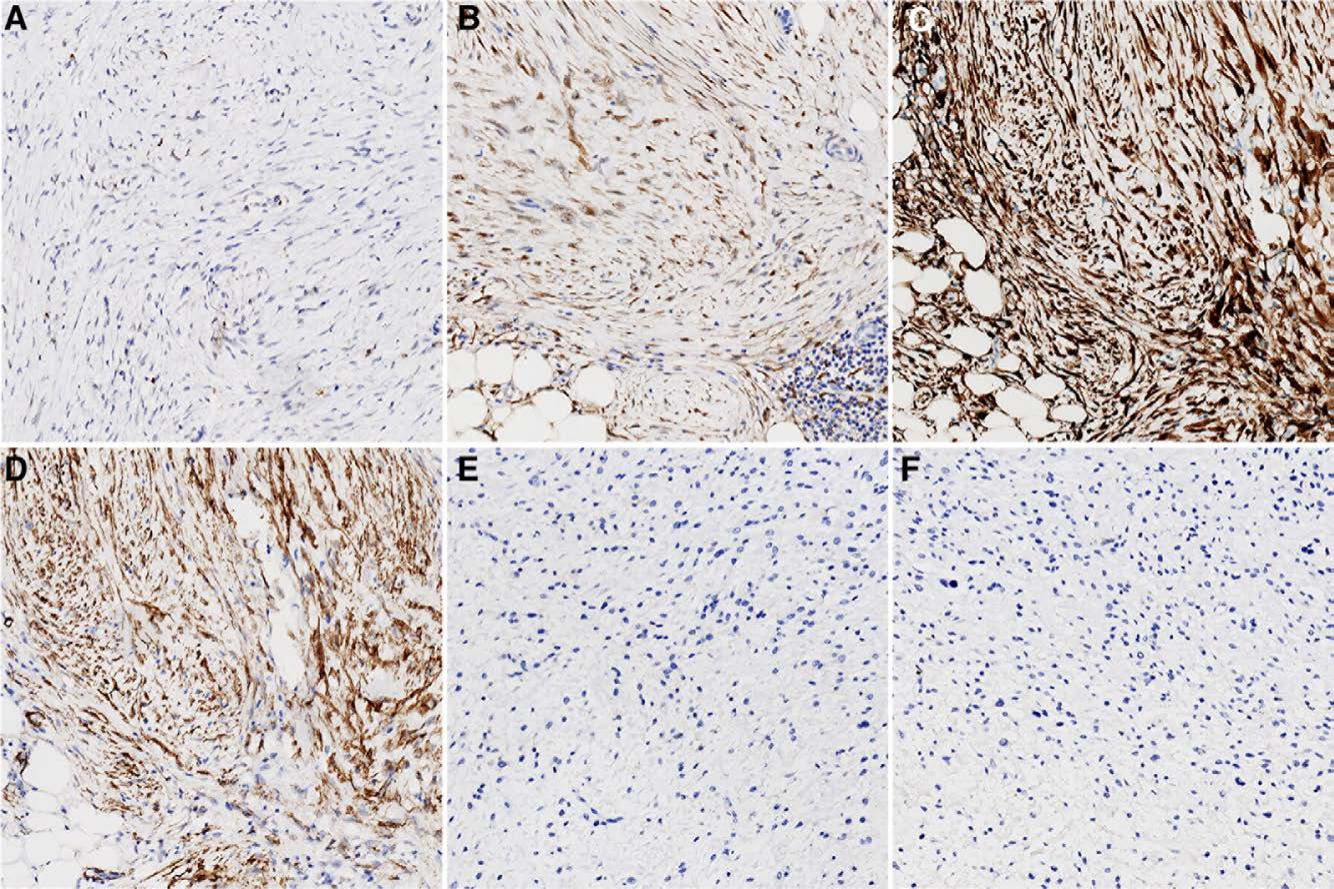
(A) Scattered MDM2 positivity, EnVision method, 100×. (B) Diffuse CDK4 positivity, EnVision method, 100×. (C) Diffuse strong P16 positivity, EnVision method, 100×. (D) Diffuse SMA positivity, EnVision method, 100×. (F) MUC4 negativity, EnVision method, 100×. (F) ALK(D5F3) negativity, EnVision method, 100×. CDK4 = cluster of differentiation kinase 4, IHC = immunohistochemistry, MDM2 = mouse double minute 2 homolog, MUC4 = mucin 4, P16 = cyclin-dependent kinase inhibitor 2A (CDKN2A), SMA = smooth muscle actin.

Immunohistochemical analysis suggested positivity for vimentin, murine double minute 2 (MDM2; Fig. 3A), and cyclin-dependent kinase 4 (CDK4; Fig. 3B), in all histological morphologies. The Ki-67 proliferation index was 20%, 10%, and 1% for the ALT/WDLPS area, LGFMS-like region, and IMT-like region, respectively. Pan-cytokeratin, desmin, β-catenin, cluster of differentiation (CD)117, discovered on gastrointestinal stromal 1 (DOG1), S100, SRY-box transcription factor 10 (SOX10), human melanoma black 45 (HMB45), MyoD1 and H-caldesmon were consistently negative in all histological morphologies. The key diagnostic discriminators reside in the differential immunoprofiles: while the IMT-like area demonstrates diffuse positivity for CDK inhibitor 2A (P16; Fig. 3C) and smooth muscle actin (SMA; Fig. 3D), it shows negativity for anaplastic lymphoma kinase (ALK; D5F3; Fig. 3F) and CD34. Conversely, LGFMS-like areas exhibit focal CD34 positivity with complete absence of mucin 4 cell surface associated protein (MUC4) expression (Fig. 3E), contrasting sharply with the IMT phenotype.

**Figure 4.**
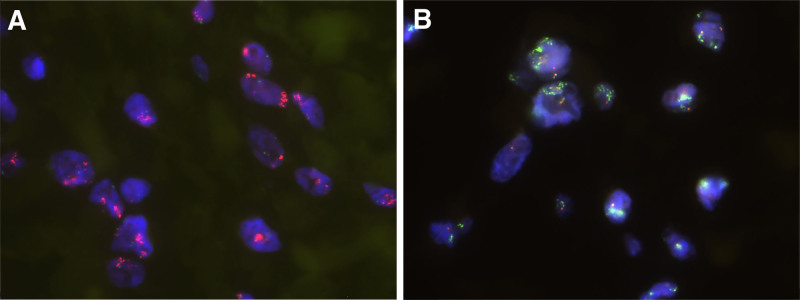
(A) MDM2 gene amplification, FISH, 1000×. (B) Absence of DDIT3 gene breakage or translocation, FISH, 1000×. DDIT3 = DNA damage-inducible transcript 3, FISH = fluorescence in situ hybridization, MDM2 = mouse double minute 2 homolog.

Fluorescence in situ hybridization (FISH) analysis indicated *MDM2* gene amplification in the dedifferentiated areas (Fig. 4A). No *DDIT3* gene rearrangement was detected in the ALT/WDLPS region with mucinous stroma (Fig. 4B). Additionally, molecular analysis confirmed the absence of characteristic genetic alterations, with no detected *FUS* gene rearrangements in LGFMS-like areas nor *ALK* gene rearrangements in IMT-like regions.

The final diagnosis was DDLPS, which consisted of components from WDLPS and DDLPS. The dedifferentiated component exhibited features resembling LGFMS and IMT, classified as FNCLCC grade 2.

After surgery, the patient did not receive adjuvant radiotherapy or chemotherapy but underwent regular monitoring with chest and abdominal computed tomography scans. At the 1-year follow-up, no recurrence or metastasis was observed.

## 3. Materials and methods

### 3.1. Histopathology and immunohistochemistry

The specimens were fixed in 10% neutral buffered formalin, dehydrated, embedded in paraffin, sectioned at 4 μm thickness, and stained with hematoxylin and eosin. Light microscopy was utilized for examination. Immunohistochemistry (IHC) was conducted using the EnVision method, incorporating high-temperature and high-pressure antigen retrieval, using 3,3′-diaminobenzidine as the chromogen. Positive controls consisted of known positive tissues, whereas negative controls consisted of PBS in place of the primary antibody. The primary antibodies used included pan-cytokeratin, vimentin, desmin, SMA, CD34, S100, SOX10, MDM2, CDK4, P16, CD117, DOG1, HMB45, MUC4, and ALK(D5F3). Different antibody combinations were selected for specific regions of histological differentiation. All kits were purchased from Fuzhou Maixin Biotechnology Development Co., Ltd.

### 3.2. FISH analysis

FISH detected *MDM2* amplification in the differentiated area (Fig. 3A), whereas no gene rearrangement of *DDIT3* was observed in the ALT/WDLPS with myxoid matrix (Fig. 3B). No *FUS* gene break-apart translocation or *ALK* gene fusion was observed in the LGFMS- and IMT-like areas, respectively. All reagents were purchased from Wuhan Kanglu Biotechnology Co., Ltd (Healthcare, Wuhan, China), and all experimental steps were conducted per the reagent protocols.

## 4. Discussion

In 1997, Henricks et al^[2]^ acknowledged that, besides high-grade sarcomas, such as pleomorphic undifferentiated sarcoma and malignant peripheral nerve sheath tumor, DDLPS can differentiate into low-grade forms resembling fibromatosis or low-grade fibrosarcomas. Furthermore, they proposed that low-grade dedifferentiation may precede high-grade dedifferentiation, with no discernible prognostic difference in prognosis between the 2 forms. In 2010, Lucas et al^[8]^ reported 6 cases of DDLPS exhibiting IMT-like histology, introducing the term “DDLPS with IMT-like features.” In 2018, Kai et al^[9]^ reported retroperitoneal DDLPS displaying IMT-like morphology. Subsequently, multiple research teams reported 18 cases, including this study.^[10–12]^ Notably, a literature search suggested 1 instance of DDLPS presenting 2 low-grade sarcomatoid morphologies, namely spinal meningioma and an inflammatory myofibroblast tumor-like structure.^[13]^ Including this case, DDLPS with IMT-like features predominantly affects middle-aged and older men (41–84 years, mean age 65.2 years; M/F = 6.5:1). The primary sites involve deep soft tissues, such as the retroperitoneum (6/18 cases), abdominal cavity (3/18 cases), inguinal/scrotal region (4/18 cases), mesentery (2/18 cases), and bowel/intestinal wall (3/18 cases). These tumors exhibit a large or multinodular morphology with fatty and nonfatty solid components, ranging from 1.3 to 20 cm. In this case, the patient, a 60-year-old man, presented with a mass involving the abdominal colonic wall/mesocolon and retroperitoneum. Including this case, 17 cases comprising distinct components of WDLPS have been recorded. Histological morphology predominantly exhibited lipoma-like ALT/WDLPS features, with a focal abundance of mucinous matrix and “chicken-foot” blood vessels, differentiating it from myxoid liposarcoma. Immunohistochemical analysis suggested MDM2, CDK4, and P16 positivity in tumor cells, with *MDM2* amplification confirmed in the mucinous area. However, *DDIT3* gene translocation was not detected, confirming it did not represent a genuine myxoid liposarcoma but a subtype within the ALT/WDLPS spectrum, that is, mucinous-type WDLPS.

In DDLPS with an IMT-like structure, similarities to classical IMT, including spindle or stellate cell distributions within a prominent loose myxoid stroma, dense fascicular or storiform arrangements of spindle cells accompanied by abundant inflammatory infiltrates, and sparsely cellular types with sclerotic matrix, are observed. Among these, the most common subtype is characterized by sparse cellularity within a sclerotic matrix.^[10]^ Morphologically, the tumor cells exhibit mild features, with vacuolated nuclei and 1 to 2 distinct nucleoli. Mitotic figures are rare, and necrosis is infrequent. SMA expression in tumor cells suggests their fibroblastic/myofibroblastic origin, leading to misdiagnosis as classical IMT. However, this case demonstrated strong positivity for MDM2, CDK4, and P16 in the IMT-like area, along with negativity for ALK(D5F3). Thus, IMT-like areas represent a dedifferentiated component rather than an IMT. FISH testing confirmed *MDM2* gene amplification and the absence of *ALK* translocation, supporting DDLPS diagnosis.

Between 2000 and 2020, the HASEGAWA^[14]^ team reported several cases of DDLPS resembling LGFMS morphology. These cases exhibited cellular arrangements of varying densities, characterized by a rich mucinous matrix. Additionally, angiogenesis with varying degrees of vessel wall sclerosis was observed. Areas with low cell density exhibited stromal collagenization, and tumor cells displayed short bundles or whirlpool-like patterns. The tumor cells exhibited mild atypia and appeared as short spindle-shaped or stellate forms, with rare mitotic figures. Immunohistochemical analysis suggested vimentin and CD34 positivity. Differential diagnosis should consider sclerosing epithelioid fibrosarcoma, myxofibrosarcoma, desmoid-type fibromatosis, and neurofibroma based on factors, such as age and the site of onset. Furthermore, MUC4 showed negativity, whereas MDM2, CDK4, and P16 showed strong positivity through IHC assessment. FISH analysis confirmed *MDM2* gene amplification without *FUS* translocation. Therefore, this case was diagnosed as DDLPS with LGFMS-like features.

In this case, the abdominal/pelvic tumor displayed a multinodular pattern, with different morphologies, immunophenotypes, and proliferation indices across the abdominal cavity, mesentery, and retroperitoneum. Complex morphological features and immunophenotypes of tumors can create confusion during diagnosis. This warrants extensive and careful sampling and observation of the tumor-fat interface for accurate diagnosis and differential diagnosis. Some scholars have reported cases^[9]^ of completely undifferentiated DDLPS components without distinct liposarcoma features, posing a diagnostic challenge and necessitating molecular testing. DDLPS and ALT/WDLPS share genetic characteristics, primarily characterized by large circular chromosomes derived from the long arm of chromosome 12(12q). These chromosomes undergo amplification in the 12q13-15 region, encompassing crucial genes, such as *MDM2, CDK4, HMGA2*, and *CPM.*^[15–17]^ Of these, *MDM2* serves as the primary driver in ALT/WDLPS and DDLPS pathogenesis, with approximately a 100% amplification rate. *CDK4* amplification is observed in approximately 85% of cases. FISH is the gold standard for detecting *MDM2/CDK4* amplification, confirmed by IHC findings within ALT/WDLPS and DDLPS regions. Furthermore, P16 is a sensitive marker, exhibiting strong, diffuse positivity within dedifferentiated areas. In this case, P16 demonstrated diffuse positivity across all examined regions. Notably, *MDM2* amplification has been identified in other soft tissue tumors, such as low-grade osteosarcoma and endometrial stromal sarcoma. Researchers have even reported immunohistochemical expression of MDM2 and CDK4 in Castleman disease.^[18]^ Therefore, challenging cases warrant a comprehensive approach integrating clinical data, pathological morphology, immunophenotype, and molecular genetic findings. Meticulous sampling is strongly recommended to enhance diagnostic accuracy. This case highlights the significant clinical implications of recognizing rare DDLPS variants. Definitive identification of dual low-grade morphologies facilitates appropriate surgical planning targeting R0/R1 margins, avoids ineffective chemotherapies given their limited overall survival benefit, and enables timely enrollment in targeted therapy trials for MDM2/CDK4 inhibition. Early detection proves particularly valuable for patients with inoperable or recurrent disease, where novel agents demonstrate therapeutic promise.

Consistent amplification of the fibroblast growth factor receptor substrate 2 (*FRS2*) gene has been reported in ALT/WDLPS with an amplification rate of 93.2%, confirming it as a novel and reliable biomarker for diagnosing ALT/WDLPS/DDLPS. Moreover, variations in the degree of *FRS2* amplification may correlate with clinicopathological characteristics. Notably, lower-grade DDLPS exhibits a higher *FRS2/CEP12* ratio, compared with ALT/WDLPS.^[19,20]^ Surgical resection remains the primary treatment modality for rare DDLPS cases transforming into low-grade sarcoma components. Postoperative adjuvant therapy, including chemotherapy and/or radiotherapy, is frequently administered. Nutlins, selective MDM2 inhibitors targeting the MDM2-p53 interaction,^[21]^ and CDK4/6 inhibitors including palbociclib (PD0332991), ribociclib (LEE011), and abemaciclib (LY2835219) show clinical promise in oncology trials. Recent findings validate their ability to induce tumor regression and promote apoptosis. Follow-up durations range from 3 to 71 months, with most patients experiencing recurrence or metastasis. Significant knowledge gaps persist concerning these rare variants: The molecular determinants underlying specific low-grade morphological patterns remain poorly understood. Prognostic distinctions between monomorphic and dual-pattern dedifferentiation phenotypes require clarification. Optimal therapeutic approaches remain undefined, particularly whether low-grade variants differ in their response to MDM2/CDK4 inhibitors compared to conventional high-grade DDLPS.

In summary, DDLPS displaying both LGFMS- and IMT-like low-grade sarcoma characteristics is exceedingly rare. Compared with LGFMS and IMT, DDLPS demonstrates more aggressive biological behavior and an unfavorable prognosis. Therefore, accurate differentiation between these entities is of utmost importance.

Looking ahead, key developments anticipated over the next 5 years include refinement of diagnostic criteria through integration of *FRS2* assessment and artificial intelligence-based pattern recognition; biology-driven classification systems correlating morphological variants with specific 12q13-15 amplifications; and morphology-stratified clinical trials evaluating CDK4/6 inhibitors combined with MDM2 antagonists. Targeted neoadjuvant strategies converting unresectable low-grade variants to operable status represent a promising therapeutic direction.

This study has methodological limitations inherent to single case reports, restricting generalizability. Molecular analysis was confined to *MDM2* amplification without comprehensive evaluation of co-amplified biomarkers (*CDK4, FRS2*) or next-generation sequencing. Multi-institutional studies with extended follow-up and multi-omics approaches are required for validation.

## Author contributions

**Writing – original draft:** Jing Huang.

**Funding acquisition:** Jing Huang, Lixia Zeng.

**Investigation:** Jing Huang.

**Methodology:** Xiaoyu Chen, Yuzhen Huang.

**Writing – review & editing:** Yun Ma.
